# Evaluation of the New FecalSwab System for Maintaining Stability of Stool Samples Submitted for Molecular Tests

**DOI:** 10.1128/JCM.00273-17

**Published:** 2017-04-25

**Authors:** Suzane Silbert, Alicia Gostnell, Carly Kubasek, Raymond Widen

**Affiliations:** Esoteric Testing/R&D Laboratory, Pathology Department, Tampa General Hospital, Tampa, Florida, USA; UNC Health Care System

**Keywords:** Biofire, FecalSwab, GI Panel

## LETTER

Gastrointestinal disease accounts for significant morbidity and mortality worldwide and may be caused by a variety of agents, including bacterial, viral, and parasitic pathogens (1, 2, 3). Rapid molecular multiplex testing has recently been introduced into enteric diagnostics and is revolutionizing patient diagnosis and treatment for diarrheal diseases (4, 5, 6, 7). Maintaining organism viability (especially the viability of bacteria) is still important for further antimicrobial susceptibility testing, when pertinent. However, inability to obtain a stool specimen at the time of the patient visit can delay the diagnostic process and contribute to inappropriate treatment (1, 2, 3, 4).

The new FecalSwab system (Copan Diagnostics, Murrieta, CA) is a convenient system for collecting rectal swab samples or for transporting fecal specimens in small instrument-ready tubes, making it easier to transport the specimen to the laboratory (8). The FecalSwab comes in a tube with 2 ml of modified Cary-Blair medium and a flocked swab. While the FecalSwab is FDA cleared for transport and culture of gastrointestinal (GI) pathogens, it is not FDA cleared for use with any molecular GI assays (9, 10). The objective of this study was to evaluate the FecalSwab for the simultaneous qualitative detection and identification of 22 GI pathogens, using a FilmArray GI Panel (Biofire Diagnostics, Salt Lake City, UT) and the FilmArray system.

A total of 103 clinical stool samples were evaluated in this study. Only one sample was included from each patient. Samples were tested by the use of a FilmArray GI Panel and the FilmArray System using two different protocols: the standard of care (SC) and the FecalSwab (FS) protocols. The SC entailed suspending fresh stool sample in Cary-Blair medium, as recommended by the FilmArray GI Panel manufacturer's protocol. Approximately 1 g of fresh clinical stool samples received in the laboratory was transferred to a screw-cap tube filled with 15 ml of Cary-Blair liquid medium (Remel, Lenexa, KS). For the FS protocol, a residual fresh stool sample was transferred to the Cary-Blair media using the provided flocked swab in accordance with the manufacturer's recommendations (11). Briefly, a small amount of fresh stool was collected by insertion of the tip of the flocked swab into the stool sample and rotation of the swab. The swab was carefully transferred into the FecalSwab tube to ensure that the swab did not exceed the filling limit indicated on the label. The vial was shaken until the sample appeared homogeneous. Fresh stool samples were transferred at the same time to the 15-ml Cary-Blair tube (SC) and to the FecalSwab Cary-Blair tube (FS). Testing on the FilmArray platform was performed according to the manufacturer's instructions (12) using 200 μl of either Cary-Blair stool samples (SC procedure) or FecalSwab transport medium (FS procedure). Results determined for the SC and FS from the same sample were compared. Additionally, a preservation/stability analysis was performed to assess the capability of the two transport media to preserve the nucleic acid in the sample. For this analysis, FS and SC stools from the first 25 positive samples were retested by the use of the FilmArray GI Panel after the samples were held for 24 h at room temperature (RT) and the results were compared to the original (0-h) results.

Of 103 patients, 33 (32%) had no pathogen detected and 70 (68%) were positive for 81 and 82 pathogens from the SC and FS protocols, respectively (Table 1). The most commonly detected pathogens included C. difficile (*n* = 31 for SC and 33 for FS), norovirus GI/GII (*n* = 17 SC and FC), and enteropathogenic Escherichia coli (EPEC) (*n* = 9 SC and FC). Eight additional pathogens (see Table 1) were detected in fewer than 6 samples.

**TABLE 1:** FilmArray GI Panel results from standard of care and fecal swab specimens

Pathogen(s) detected^*a*^	No. of specimens	
	Standard of care (SC)	Fecal Swab (FS)
Clostridium difficile toxin A/B	31	33
Campylobacter	3	3
Plesiomonas shigelloides	1	1
Salmonella	2	2
Yersinia enterocolitica	1	1
EPEC	9	9
ETEC	4	4
EIEC	1	1
Cryptosporidium	3	2
Astrovirus	1	1
Norovirus GI/GII	17	17
Rotavirus A	3	3
Sapovirus	5	5
Total	81	82

a EPEC, enteropathogenic Escherichia coli; ETEC, enterotoxigenic E. coli *stx*_1_/*stx*_2_; EIEC, Shigella/enteroinvasive E. coli.

For 33 negative and 67 positive samples, there was complete agreement between the SC and FS testing protocols (overall agreement = 97.1%, 100/103 samples). Three samples had discrepant results; two were positive for C. difficile only when tested using the FS protocol and one was positive for Cryptosporidium only when tested using the SC protocol. The three samples with discrepant results were tested after 24 h of room temperature storage. C. difficile was detected in both samples when tested using the FS and SC protocols (Table 2, samples 9 and 10). Conversely, the FilmArray GI Panel result for Crytosporidium was negative after 24 h of storage using both the SC and FS protocols (Table 2, sample 25).

**TABLE 2:** Results from preservation/stability analysis of 25 positive samples evaluated

Sample(s) tested	Result for^*a*^:			
	SC at 0 h	FS at 0 h	SC at 24 h	FS at 24 h
1–8	C. difficile	C. difficile	C. difficile	C. difficile
9–10	Negative	C. difficile	C. difficile	C. difficile
11–13	Norovirus GI/GII	Norovirus GI/GII	Norovirus GI/GII	Norovirus GI/GII
14–15	Salmonella	Salmonella	Salmonella	Salmonella
16–17	EPEC	EPEC	EPEC	EPEC
18	Cryptosporidium	Cryptosporidium	Cryptosporidium/norovirus	Cryptosporidium/norovirus
19	ETEC/EIEC	ETEC/EIEC	ETEC/EIEC	ETEC/EIEC
20	Campylobacter/C. difficile	Campylobacter/C. difficile	C. difficile	C. difficile
21	C. difficile/norovirus	C. difficile/norovirus	C. difficile/norovirus	C. difficile/norovirus
22	EPEC/astrovirus	EPEC/astrovirus	EPEC/astrovirus	EPEC/astrovirus
23	EPEC/ETEC	EPEC/ETEC	EPEC/ETEC	EPEC/ETEC
24	Sapovirus	Sapovirus	Sapovirus	Sapovirus
25	EIEC/Cryptosporidium	EIEC	EIEC	EIEC

a EPEC, enteropathogenic Escherichia coli; ETEC, enterotoxigenic E. coli *stx*_1_/*stx*_2_; EIEC, Shigella/enteroinvasive E. coli.

Of the 25 samples tested in the preservation/stability analysis, 20 of the samples processed using the SC protocol and 23 of the samples processed using the FS protocol had concordant results at the 0-h and 24-h time points (Table 2). Of the samples processed using the SC protocol, 2 were positive at the 0-h time point but negative at the 24-h time point (Table 2, sample 20 [Campylobacter] and sample 25 [Cryptosporidium]) and 3 had new detection at the 24-h time point (Table 2, samples 9 and 10 [C. difficile] and sample 18 [norovirus]). For samples processed using the FS protocol, one sample was initially positive but retested negative (Table 2, sample 20 [Campylobacter]) and one sample had a new detection at the 24 time point (Table 2, sample 18 [norovirus]). Two of discrepancies were observed in the same samples by both protocols tested (Table 2, samples 18 and 20). Data files from the five discrepant samples were sent to BioFire Diagnostics for further analysis. In all cases, the discrepant results showed evidence of late amplifications indicating a relatively low pathogen concentration that was below the concentration that was reliably detected by the corresponding FilmArray GI Panel assay. Detection of additional pathogens after extended incubation of the samples could be also explained by organism growth during the storage period; however, Cary-Blair transport medium is designed for the preservation of gastrointestinal pathogens and is not supposed to support organism growth.

Studies have indicated that rectal swab specimens (13, 14, 15), and more recently, Copan FecalSwab specimens (8, 9, 10) can provide accurate test results when used with stool culture and molecular testing of enteric pathogens. Our study results corroborate those of previous studies. Overall, the performances of the 0-h and 24-h FecalSwab specimen PCRs observed in this study were equivalent to those seen using traditional Cary-Blair specimens for detection of GI pathogens using the FilmArray GI Panel on the FilmArray system. The rate of discrepant results observed between the SC and FS procedures was less than 3%. The discordant results were most likely the consequence of a low organism concentration; however, they could also have been caused by weak cross-reactivity and/or contamination during the test process. To conclude, our data support the idea that the FecalSwab system can be used to process raw stool samples prior to testing with the FilmArray GI Panel. Our study, however, did not address the use of the FilmArray GI Panel with rectal swabs. The FecalSwab system optimizes the collection and transport of GI pathogens and rapid diagnosis of gastrointestinal diseases.
